# Accuracy of using computer-aided rapid prototyping templates for mandible reconstruction with an iliac crest graft

**DOI:** 10.1186/1477-7819-12-190

**Published:** 2014-06-24

**Authors:** Da-long Shu, Xiang-zhen Liu, Bing Guo, Wei Ran, Xin Liao, Yun-yan Zhang

**Affiliations:** 1 Department of Oral and Maxillofacial Surgery, the First Affiliated Hospital of Sun Yat-sen University, No. 58 Zhongshan Er Road, Guangzhou 510080, China

**Keywords:** Mandibular tumor, Mandible reconstruction, Virtual surgical planning

## Abstract

**Background:**

This study aimed to evaluate the accuracy of surgical outcomes in free iliac crest mandibular reconstructions that were carried out with virtual surgical plans and rapid prototyping templates.

**Methods:**

This study evaluated eight patients who underwent mandibular osteotomy and reconstruction with free iliac crest grafts using virtual surgical planning and designed guiding templates. Operations were performed using the prefabricated guiding templates. Postoperative three-dimensional computer models were overlaid and compared with the preoperatively designed models in the same coordinate system.

**Results:**

Compared to the virtual osteotomy, the mean error of distance of the actual mandibular osteotomy was 2.06 ± 0.86 mm. When compared to the virtual harvested grafts, the mean error volume of the actual harvested grafts was 1412.22 ± 439.24 mm^3^ (9.12% ± 2.84%). The mean error between the volume of the actual harvested grafts and the shaped grafts was 2094.35 ± 929.12 mm^3^ (12.40% ± 5.50%).

**Conclusions:**

The use of computer-aided rapid prototyping templates for virtual surgical planning appears to positively influence the accuracy of mandibular reconstruction.

## Background

Surgeons often have difficulty achieving functional and aesthetic mandibular reconstructions after ablative tumor surgery [1-4]. Traditionally, surgeons have used their past experience to determine the best way to perform the osteotomy, graft harvesting, and graft shaping procedures for mandibular reconstruction. However, computer-aided (virtual) surgical planning and rapid prototyping (RP) now offer more effective and predictable reconstruction outcomes [5], and a series of successful studies have emerged as surgeons and RP engineers have begun to cooperate [6,7]. Nevertheless, researchers are still wary about the accuracy of virtual surgical planning as well as donor-site morbidity. Therefore, the aim of our study was to evaluate the benefits of computer-assisted mandibular reconstruction with free iliac crest bone grafts regarding accuracy and the amount of bone loss. We present cases of mandibular reconstruction that employed virtual surgical planning, and for which we determined the accuracy of the actual reconstructions as compared to the virtual surgical plans. We also assessed how much tissue was harvested for the graft, with the goal being to take the least amount of tissue required.

## Methods

### Patients

This study was approved by the local ethics committee at Sun Yat-sen University, China. It was carried out after institutional approval of ethics committee of the First Affiliated Hospital of Sun Yat-sen University and written informed consent was obtained. The study included eight patients with ameloblastoma involving one side of the mandible. The study participants (5 males and 3 females; 19 to 54-years-old, mean = 30.6 years) underwent the osteotomy and sequential mandibular reconstruction using free iliac grafts between September 2008 and June 2012 at the Department of Oral and Maxillofacial Surgery, The First Affiliated Hospital, Sun Yat-sen University, China.

### Surgical planning procedure

In each case, a computed tomography (CT) scan was performed (Aquilion64 CT, Toshiba, Tochigi, Japan; slice thickness = 0.5 mm) in the mandibular, maxillary, and skull base regions, as well as on the pelvis where we planned to harvest iliac crest. The scan data was imported as standard DICOM (Digital Imaging and Communications in Medicine) files to Mimics software (Materialise, Leuven, Belgium).

**Figure 1:**
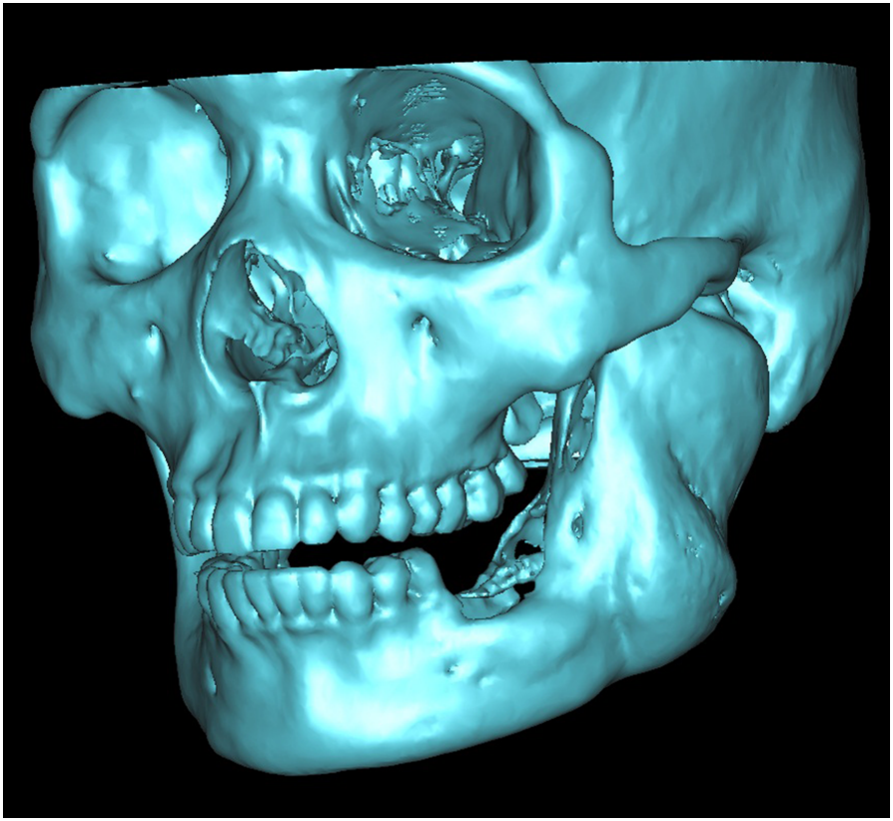
A clear view of the ameloblastoma-affected region of the mandible.

**Figure 2:**
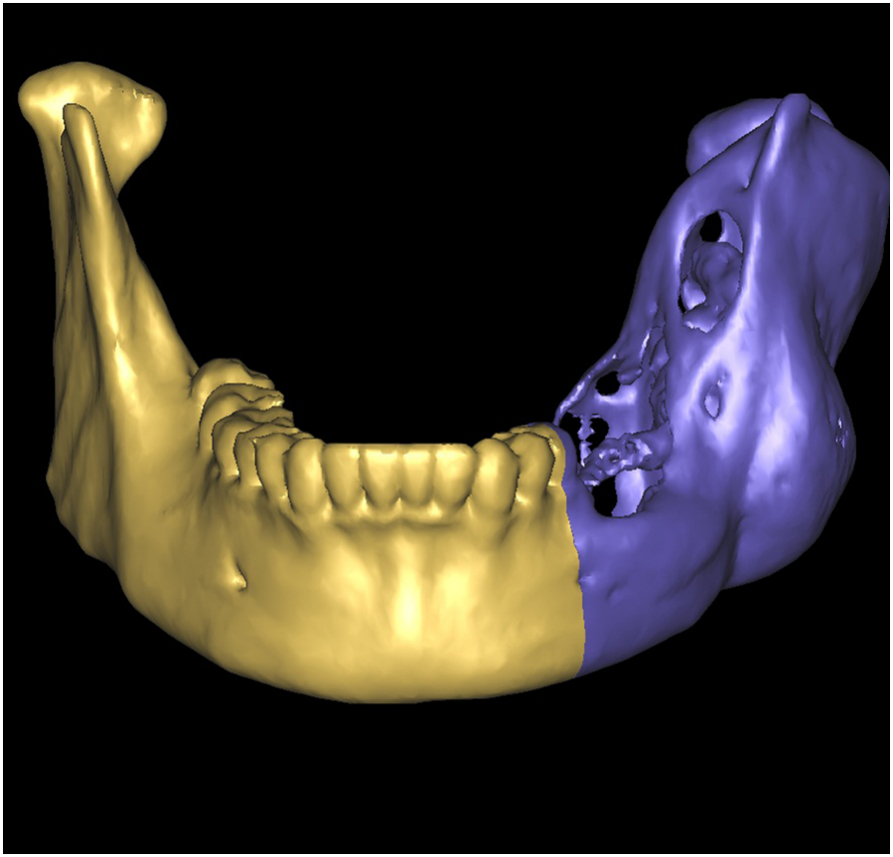
The virtual osteotomy of the affected mandible was performed on the software.

**Figure 3:**
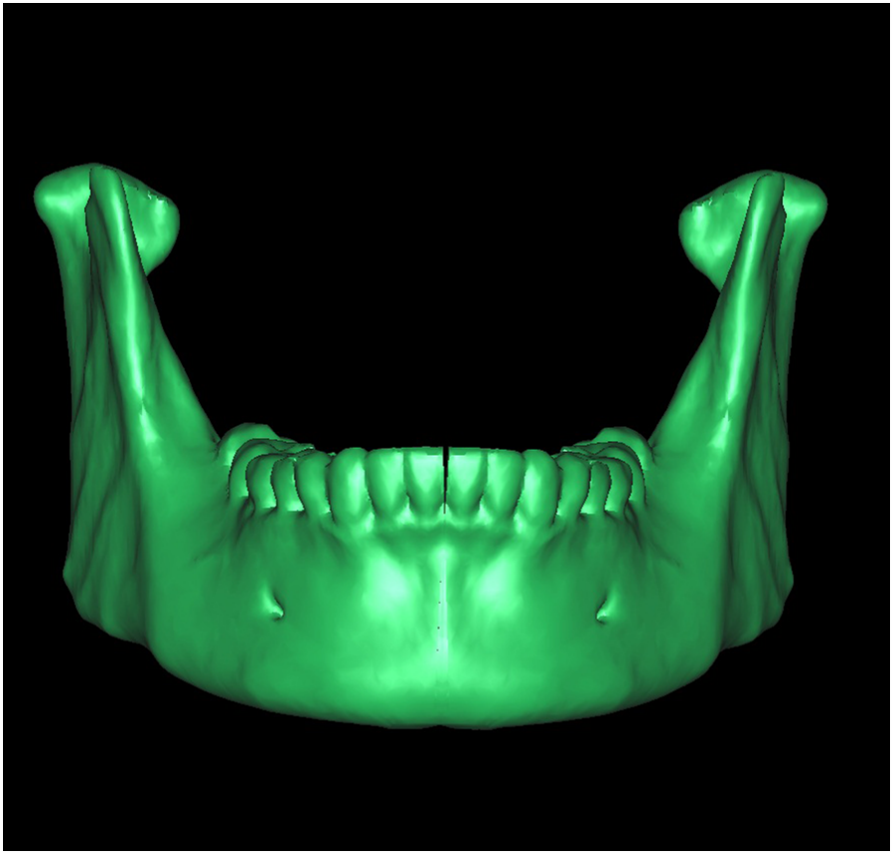
The unaffected side of mandible was reflected (mirror-duplicated) onto the resected side to restore the original mandibular contours.

**Figure 4:**
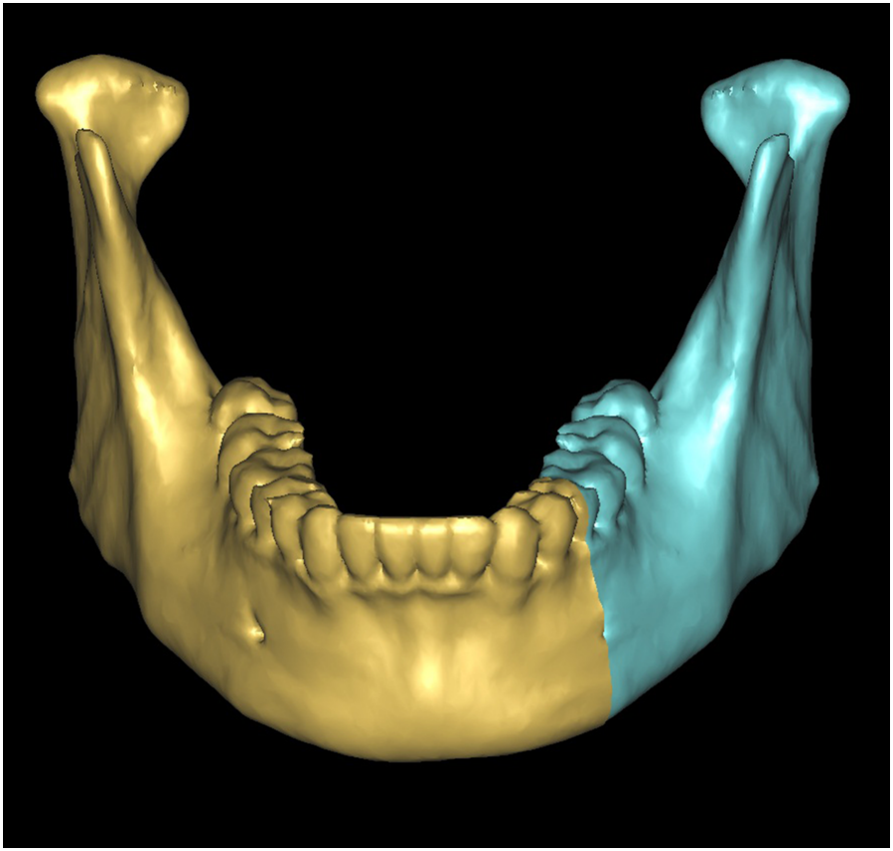
After repeating the virtual osteotomy on the mirror-duplicated mandible, we obtained the contours of virtually reconstructed mandible.

With thresholding and three-dimensional reconstructive processes, we imaged only the bone tissue to obtain a clear view of the ameloblastoma-affected region of the mandible (Figure 1). Using the software we performed a virtual osteotomy of the affected mandible (Figure 2). Then a middle sagittal plane was determined by the sella, nasion, and subspinale points [3]. Upon this plane the image of unaffected side of mandible was reflected onto the resected side to restore the original mandibular contours (Figure 3). After repeating the virtual osteotomy on the reflected image (the mirror-duplicated image) of the mandible, we determined the contours of the virtually reconstructed mandible (Figure 4).

**Figure 5:**
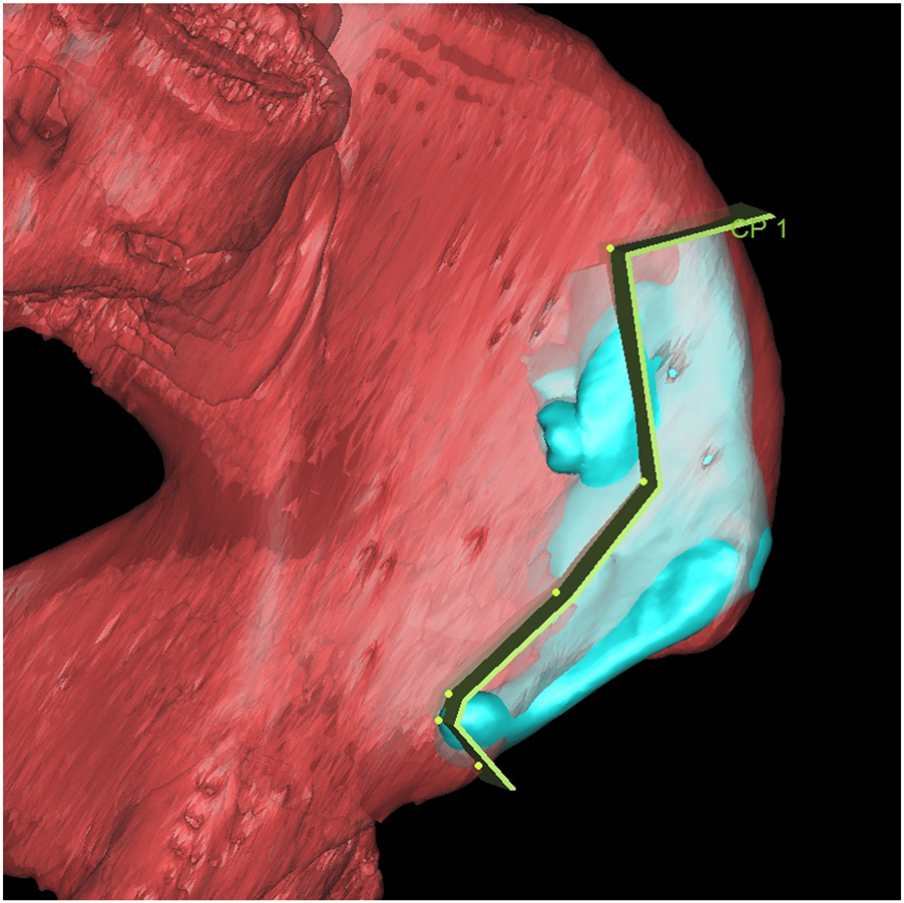
The virtually reconstructed mandible graft was overlapped with an image of the pelvis to mark where and how much iliac crest should be harvested.

**Figure 6:**
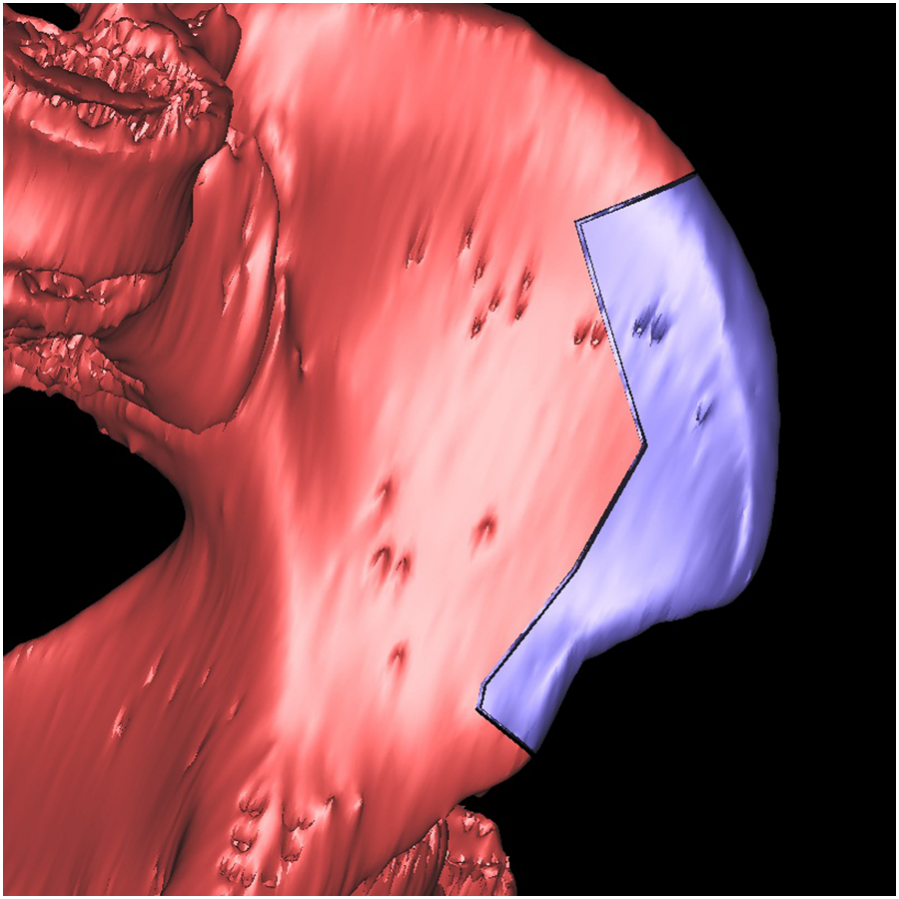
The virtual harvesting graft on the iliac crest.

Using the same procedures an image of the pelvis was isolated from the soft tissue and we three-dimensionally reconstructed it. Then we overlapped the virtually reconstructed mandible graft with the pelvis image to mark where and how much iliac crest should be harvested (Figure 5). Using these marks, we performed virtual harvesting on the iliac crest (Figure 6).

### Rapid prototyping (RP) template design

**Figure 7:**
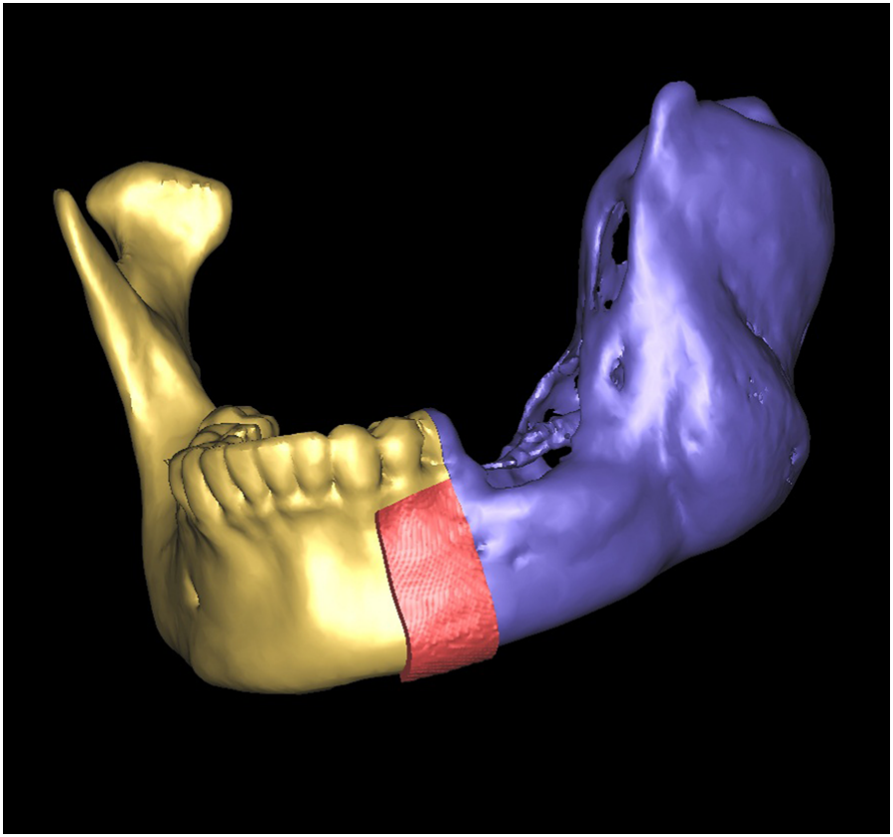
An osteotomy template accurately covered the inferior border of the mandible as well as the anterior osteotomy margins to indicated the osteotomy line.

**Figure 8:**
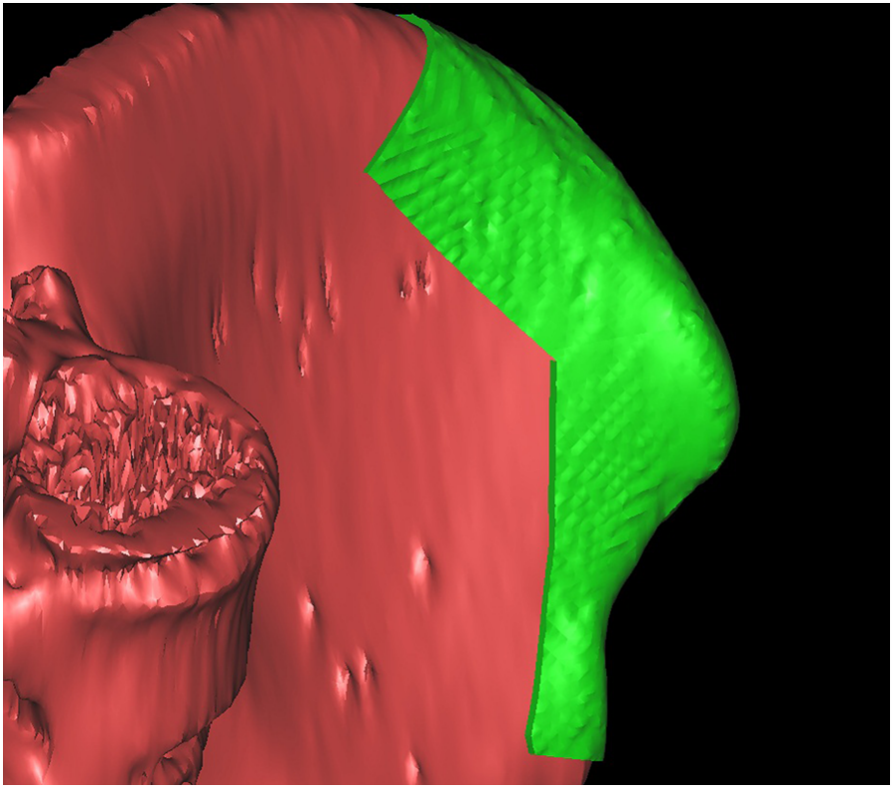
A harvesting template was designed to cover the planned harvesting region of the iliac crest.

**Figure 9:**
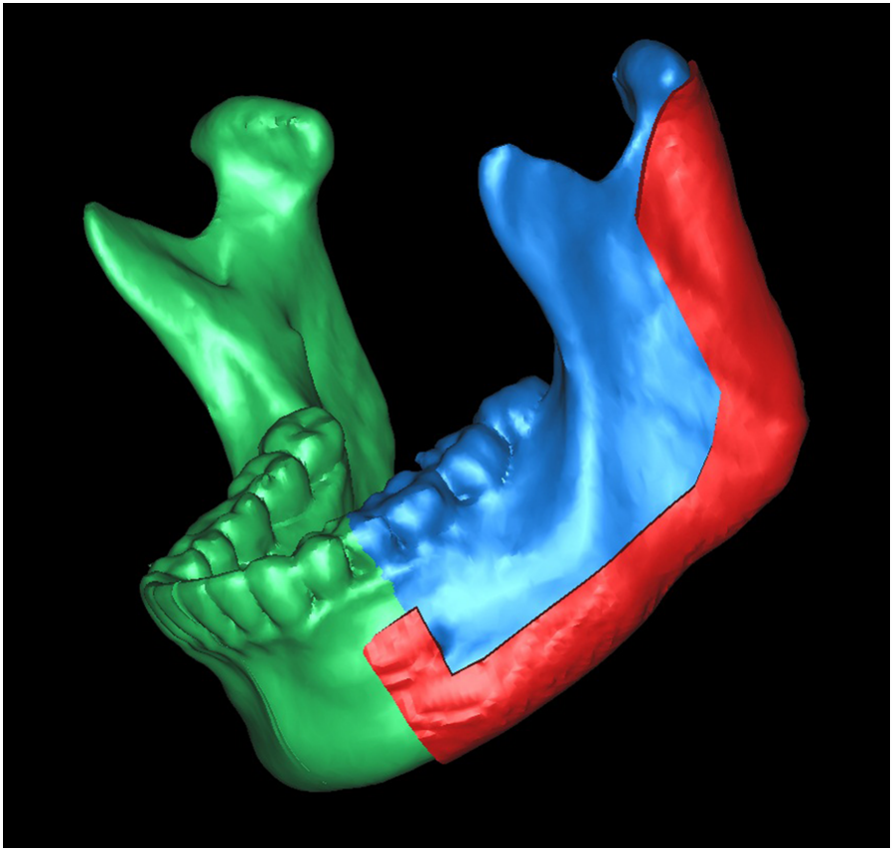
A shaping template was designed so that it exactly covered the surface of the virtually reconstructed mandible and was used to maintain the mandibular position during the operation and guide the graft into the proper position.

We used the following procedures to design RP templates, which we used to perform the operations as planned. The virtual mandible was saved as a stereolithography (STL) file. It was imported into Magics software (Materialise; Leuven, Belgium) and was duplicated using the ‘copy part’ and ‘paste part’ commands. We selected one copy, marked the surface of the bone, and then extruded the surface by 2 mm (parameter: offset 2 mm, connection automatic). We then performed Boolean operations to subtract the unchanged part from the extruded one, thereby creating a 2-mm thick shell that covered the whole mandible. We clipped the shells by removing the unnecessary parts to create custom-designed templates. The osteotomy templates accurately covered the inferior border of the mandible and indicated the osteotomy line (Figure 7). Using the same methods, we designed the harvesting templates to cover the planned harvesting region of the iliac crest and designed the shaping templates to exactly cover the surface of the virtually reconstructed mandible (Figures 8 and 9).

All designed templates were saved as STL files and sent to a fully automated rapid stereolithography machine (SLA3500, 3D Systems, Texas, United States) to fabricate RP templates. The final acrylic templates were duplicated from RP models.

### Surgical procedure

**Figure 10:**
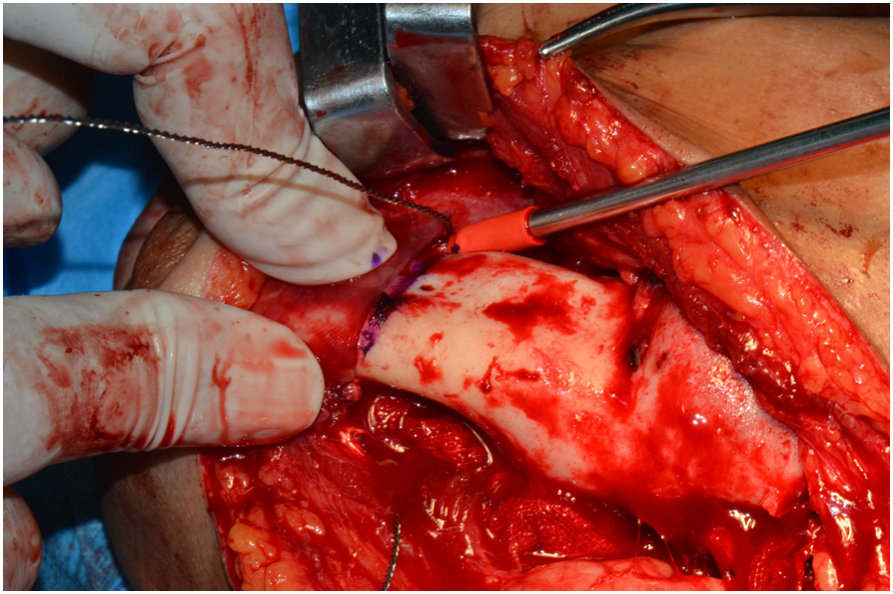
An osteotomy template was mounted on the buccal side and inferior border of the mandible and an osteotomy line was drawn according to the template.

**Figure 11:**
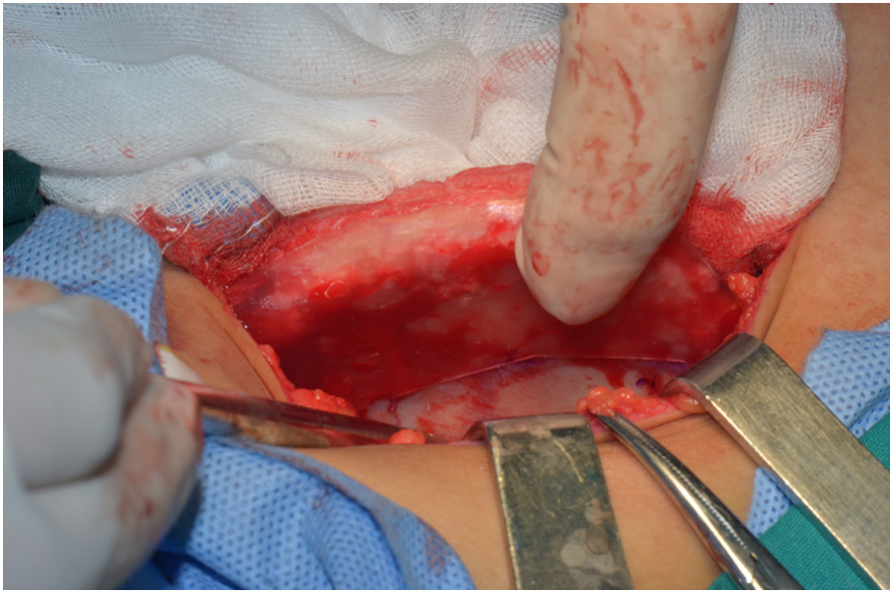
A harvesting template was mounted on the surface of the iliac crest and an osteotomy line was drawn based on the template.

**Figure 12:**
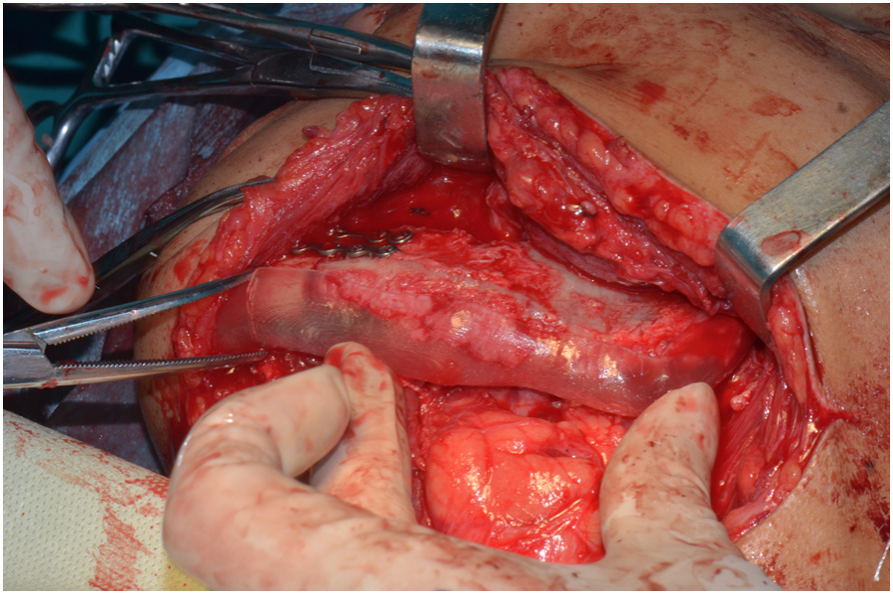
Using a shaping template, the iliac graft was shaped and placed into the proper position.

Surgeries were performed by accessing the inferior border of mandible. The osteotomy templates were mounted on the buccal side of mandible as well as on the inferior border. According to the templates, the surgeons used a marker to draw the planned osteotomy lines on the mandible surface, then resected the affected mandibles (Figure 10).Similar to the mandible, the harvesting template was mounted on the surface of the iliac crest. The harvesting margins were drawn according to the template’s outline (Figure 11). After harvesting, the iliac graft was shaped so that it matched the shaping template, and, using the shaping template as a guide, the iliac graft was placed in the proper position and rigidly fixed with the remaining mandible (Figure 12).

### Evaluation

**Figure 13:**
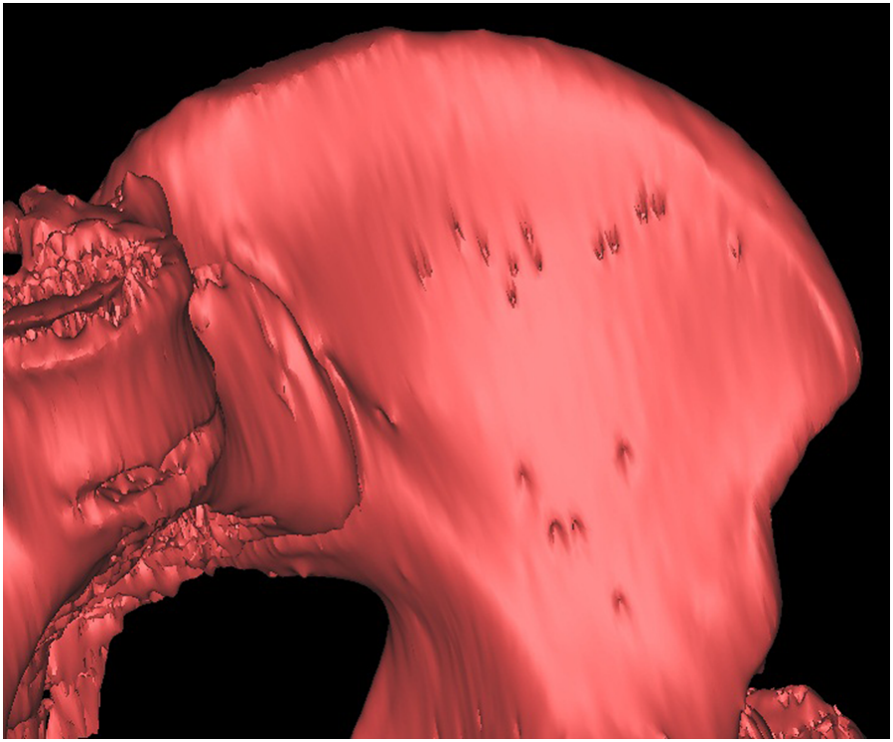
The preoperative iliac CT scan.

**Figure 14:**
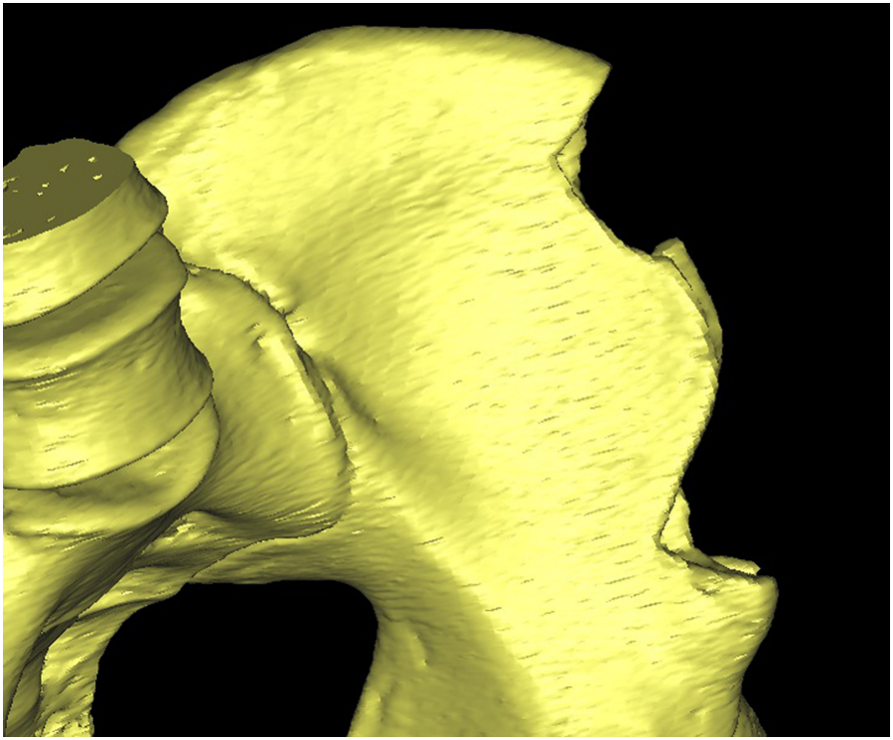
The postoperative iliac CT scan.

**Figure 15:**
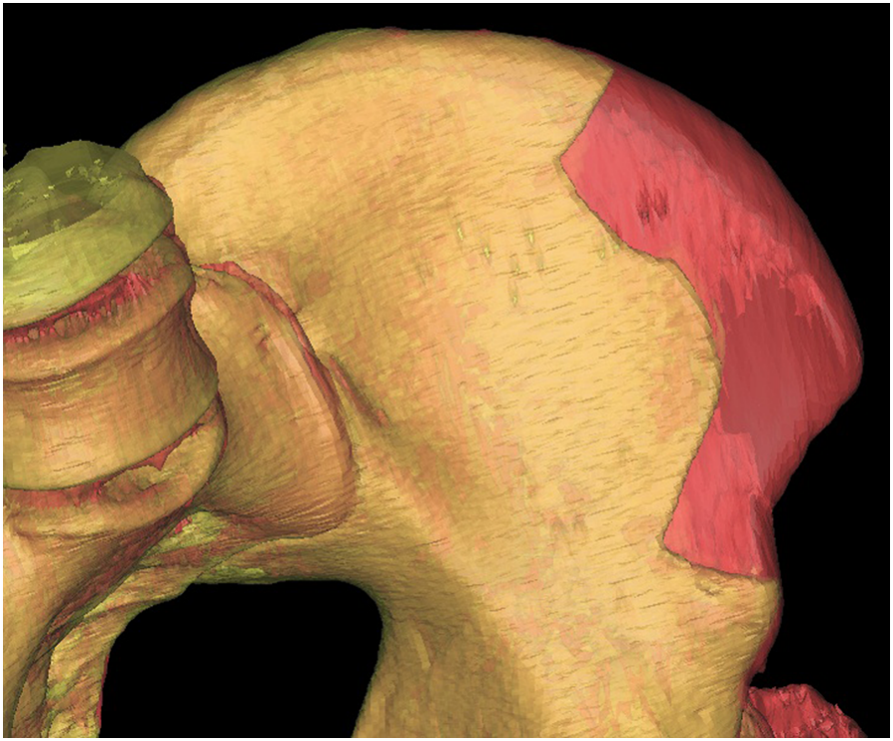
**Overlapping pre- and postoperative iliac CT scans.** The red part shows the volume of the harvested graft.

**Figure 16:**
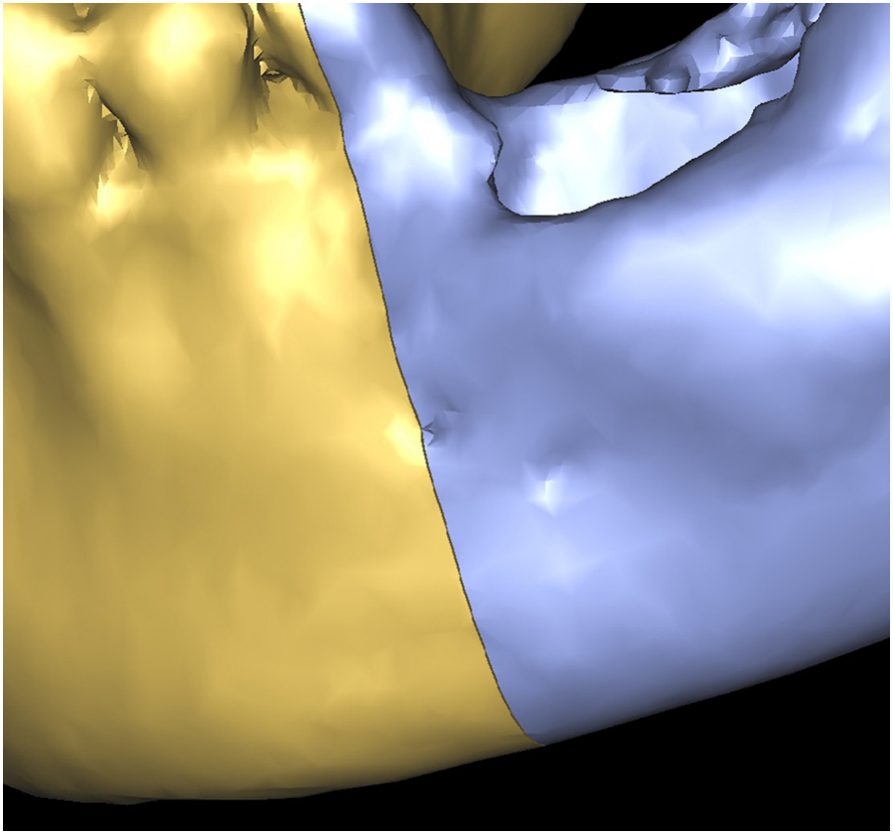
The virtual osteotomy line in the preoperative plan.

**Figure 17:**
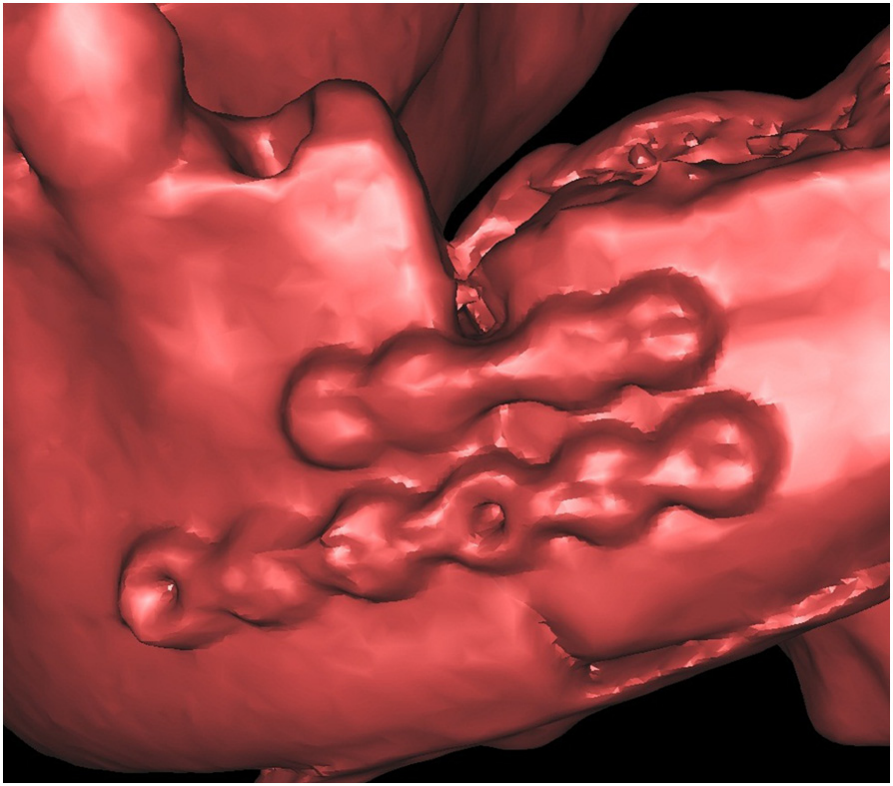
The postoperative CT scan of the reconstructed mandible.

**Figure 18:**
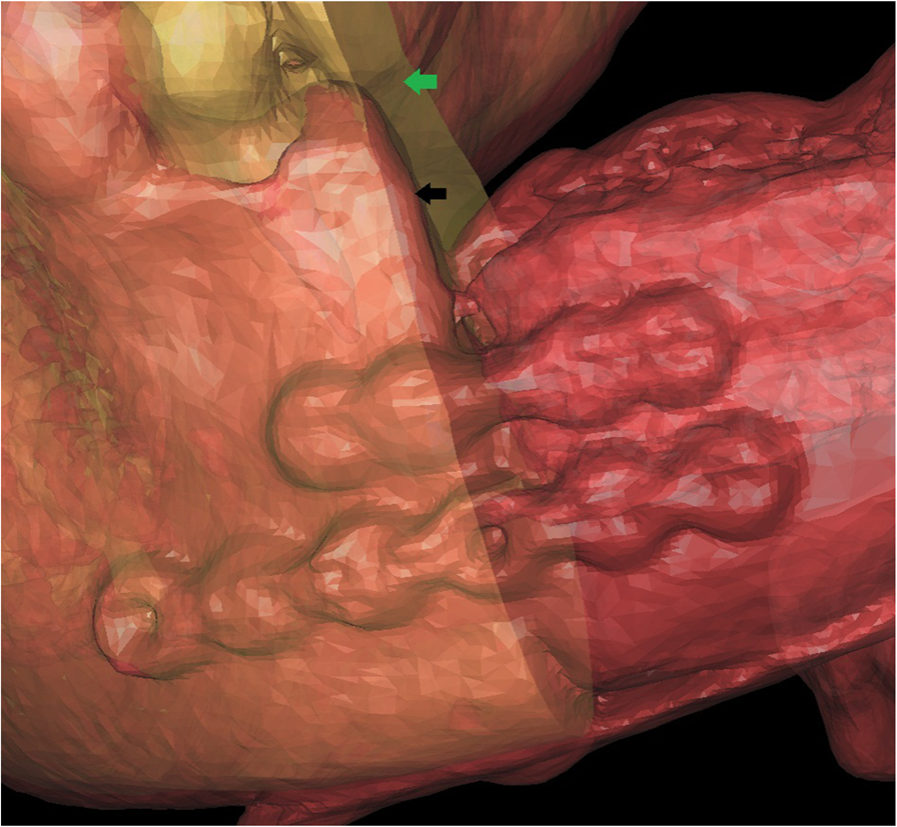
**Overlapping pre- and postoperative mandibles.** Error was measured between the virtual osteotomy line (green arrow) and the actual osteotomy line (black arrow).

**Figure 19:**
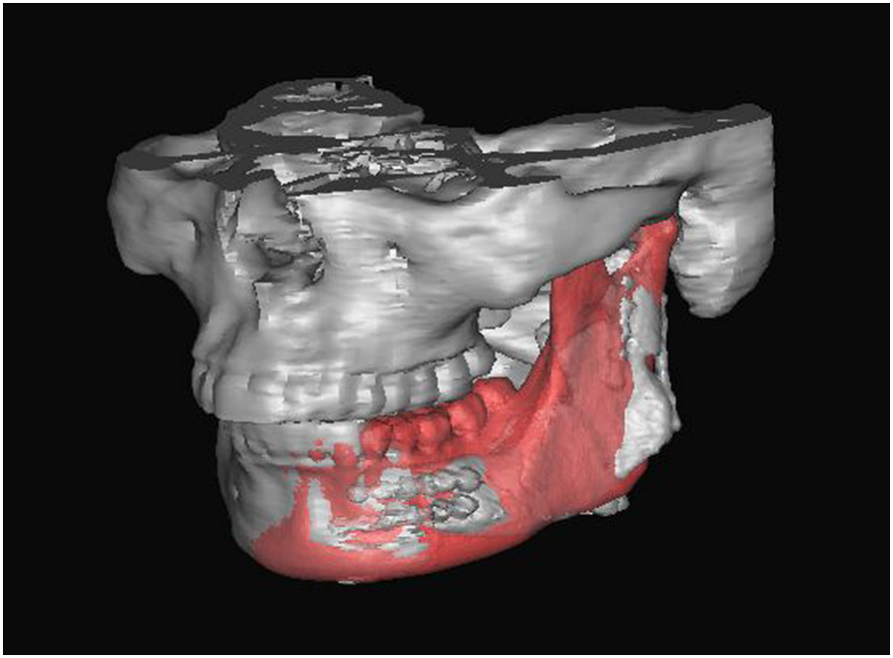
Overlapping preoperative virtually reconstructed (red) and postoperative mandibles (grey) to show the differences between the preoperative design model and graft after shaping. It shows the differences are well distributed around mandible.

A postoperative CT scan of each patient was obtained within 14 postoperative days. The postoperative three-dimensional computer models overlapped with the correlating preoperative design models in the same coordinate system (Figures 13, 14, 15, 16, 17, 18 and 19). We compared the preoperative design models and the postoperative models by measuring the distance of the mandibular osteotomy, the volume of harvested graft, and the volume of the graft after shaping. Because the eight patients in this study had ameloblastoma on only one side of the mandible, the unaffected side was used as the reference. Since the unaffected side of the mandible was reflected onto the affected side as in the virtual reconstruction, the reference is represented with the term ‘virtual’ in Table 1.

**Table 1:** Accuracy of postoperative results compared with virtual surgical planning

**No.**	**Gender/age (years)**	**Affected region**	**Distance of mandibular osteotomy (mm)**			**Volume of reconstructive graft (mm**^**3**^**)**				
			**Virtual**	**Actual**	**Error (%)**	**Virtual**	**Harvested (non-shaped)**	**Shaped**	**Error between virtual and harvested (%)**	**Error between harvested and shaped (%)**
1	F/42	MRC	109.42	107.36	-2.06 (1.88%)	27505	29525.35	25918.03	2020.40 (7.35%)	3607.32 (12.22%)
2	M/54	PM	30.75	32.49	1.74 (5.66%)	7070.37	7676.48	6590.79	606.11 (8.57%)	1085.69 (14.14%)
3	M/20	MR	57.6	59.04	1.44 (2.50%)	13018.3	14534.42	13235.51	1516.16 (11.65%)	1298.91 (8.94%)
4	F/26	MRC	101.38	102.48	1.1 (1.09%)	19253.2	20806.81	18334.37	1553.59 (8.07%)	2472.44 (11.88%)
5	M/26	M	32.18	34.8	2.62 (8.14%)	7602.38	8805.98	7588.97	1203.6 (15.83%)	1217.01 (13.82%)
6	F/36	PMR	46.65	45.31	-1.34 (2.87%)	16718.9	18267.97	16070.04	1549.11 (9.27%)	2197.93 (12.03%)
7	M/22	MRC	110.02	106.32	-3.7 (3.36%)	20917.7	22686.75	19561.82	1769.01 (8.46%)	3124.93 (13.77%)
8	M/19	PM	39.35	41.85	2.5 (6.35%)	11739.6	12819.41	11068.84	1079.83 (9.20%)	1750.57 (13.66%)
Average	30.6 ± 12.4		65.92 ± 35.08	66.21 ± 33.42	2.06 ± 0.86 (3.13% ± 1.31%)	15478.17 ± 6997.65	16890.40 ± 7395.49	14796.05 ± 6504.39	1412.22 ± 439.24 (9.12% ± 2.84%)	2094.35 ± 929.12 (12.40% ± 5.50%)

M, molar; MR, molar/ramus; MRC, molar/ramus/condyle; PM, premolar/molar; PMR, premolar/molar/ramus; -, indicates that the actual distance or volume errors were negative (errors not marked by ‘-’ mean that the actual distance or volume errors were positive).

## Results

All eight mandibular reconstructions were carried out successfully. The mean error between the distance of the actual mandibular osteotomy and the distance of the virtual osteotomy was 2.06 ± 0.86 mm. The mean error of the volume of the actual harvested grafts compared to that of the virtual harvested grafts was 1412.22 ± 439.24 mm^3^ (9.12% ± 2.84%). The mean error between the volume of the actual harvested grafts and that of the shaped grafts was 2094.35 ± 929.12 mm^3^ (12.40% ± 5.50%) (Table 1).

## Discussion

This study indicates that computer-aided rapid prototyping templates can help surgeons perform accurate operations. The error between virtual surgical planning and the actual results are acceptable and the surgeons who participated in the surgical planning and template design felt more familiar with, and confident in, the operation procedures.

Iliac grafting is a common method of mandibular reconstruction [8]. When surgeons plan to harvest such grafts they must consider the reconstruction effects, the graft survival rate, and the donor-site morbidity. The reconstruction effect is directly determined by the graft, which should allow for a symmetric facial contour and should fit well with the upper jaw. The survival rate of iliac grafts significantly correlates to the time they spend *in vitro* before the pedicled grafts are anastomosed to the recipients. The donor-site morbidity that occurs after iliac grafts are harvested, such as postoperative functional problems and pain at the donor site, mainly depend on how much bone is harvested [9]. In our study, the contour and position of the virtually reconstructed mandibles, which were designed using the mirror duplication technique, are capable of producing excellent postoperative results in terms of occlusion and symmetrical facial contouring. Furthermore, the harvesting templates allowed for more accurate harvesting and less modification to the grafts. Finally, we found that shaping templates are reliable references that can be used to quickly shape grafts, allowing surgeons to decrease the time that grafts spend *in vitro*, as well as minimize unnecessary donor-site invasion.

The accuracy of our design was previously proven when we successfully removed foreign bodies from the skull base [10]. When we applied this method to designing mandible templates we found similar results to another report [11] which evaluated the accuracy of free fibula mandibular reconstruction. We believe that the differences between the studies due to how the templates were designed are of little significance.

The mean difference between the actual and virtual harvested grafts was 9.12% ± 2.84%, and it was concurrent with Roser’s results [11] which indicate that regardless of where grafts are harvested from, controlling the accuracy of the graft harvesting procedure is always difficult. When designing templates for graft harvesting our aim was to use a minimal harvesting volume and take it from the proper location. At the same time, surgeons were reminded that the reconstructions would probably fail if they harvested less bone than the templates called for. As such, surgeons generally harvested grafts that were slightly bigger than the indicated regions. However, we believe that the more surgeons trust the guiding templates the higher the harvesting accuracy will be.

After shaping the grafts, we found that the grafts lost a mean volume of 2094.35 ± 929.12 mm^3^ (12.40% ± 5.50%). Therefore, the shaping templates allowed us to keep the loss rate of graft volume below 15%. We consider this percentage to represent an unnecessary injury that causes more donor-site morbidity. Several factors contribute to this loss rate: (1) The position where the harvesting template was mounted likely varied a little because of the surrounding soft tissue; (2) The harvesting margins drawn according to the template’s outline might have been enlarged because it was difficult to keep the marking pen vertical to the template; and (3) The reconstructive grafts were often cut low to reduce the suture tension.

Ayoub *et al.*[12] previously evaluated computer-assisted mandibular reconstruction with vascularized iliac crest bone grafts as compared to conventional surgery. In their study, conventional surgery outcomes were clinically acceptable but had a mean error of 20% between the defect size (83.3 ± 18.7 mm) and the transplant size (which significantly exceeded the defect size by 16.8 ± 5.6 mm) [12]. In our study, we found a mean error of only 12.40% between the volume of the defect size and that of the transplant size, which proves that computer-assisted mandibular reconstruction is capable of improving the accuracy of surgical outcomes and reducing donor-site morbidity.

We must mention that bone volume measurements obtained by software vary widely according to the thresholding parameters and the three-dimensional calculating quality one chooses. Thus, comparing software-measured graft volumes between different studies is meaningless. In our study, the software parameter was fixed for each case, however, considering that it is impossible for every researcher to use the same software parameters, we recommend using percentages to standardize comparisons.

Despite obtaining satisfactory outcomes we still found errors in distances and volumes when the treatment results were evaluated [13]. Various factors likely contributed to these errors. First, operative errors can be decreased but they can never be eliminated completely. Second, a slight distortion exists in the CT scan model [14,15]. Third, computer-assisted planning processes and validation processes have slight errors as well [16]. Finally, the templates might be distorted when they are fabricated.

Our study was limited by the fact that templates cannot be modified once they are fabricated. When the affected region is unclear in preoperative CT images, such as because of osteosarcoma and radioactive osteomyelitis, positive margins might be found intraoperatively. Surgeons then must make second, wider resections, and the sequential templates are then useless. Therefore, we suggest that surgeons beware of this variation and handle it with care when planning surgical procedures.

## Conclusions

The use of computer-aided rapid prototyping templates for virtual surgical planning appear to positively influence the accuracy of mandibular reconstruction.

## Competing interests

The authors declare that they have no competing interests.

## Authors’ contributions

DLS designed the study and drafted the manuscript. XZL participated in the rapid prototyping template design. WR participated in the surgical procedure. BG participated in the records collection and analysis. XL helped the surgical procedure. YYZ helped to draft the manuscript. All authors read and approved the final manuscript.

## References

[B1] Li JS Chen WL Huang ZQ Zhang DM Pediatric mandibular reconstruction after benign tumor ablation using a vascularized fibular flap J CraniofacSurg 2009 20 431 434 10.1097/SCS.0b013e31819b96db 19218857

[B2] Hallermann W Olsen S Bardyn T Taghizadeh F Banic A Iizuka T A new method for computer-aided operation planning for extensive mandibular reconstruction PlastReconstrSurg 2006 117 2431 2437 10.1097/01.prs.0000219076.83890.e8 16772952

[B3] Z'Graggen M Schiel HJ Kunz C Lambrecht JT Three-dimensional cephalometry using individual skeletal laser technology models ClinAnat 2001 14 258 268 10.1002/ca.1044 11424200

[B4] Goh BT Lee S Tideman H Stoelinga PJ Mandibular reconstruction in adults: a review Int J Oral Maxillofac Surg 2008 37 597 605 10.1016/j.ijom.2008.03.002 18450424

[B5] Goiato MC Santos MR Pesqueira AA Moreno A dos Santos DM Haddad MF Prototyping for surgical and prosthetic treatment J CraniofacSurg 2011 22 914 917 10.1097/SCS.0b013e31820f7f90 21558926

[B6] Feng F Wang H Guan X Tian W Jing W Long J Tang W Liu L Mirror imaging and preshaped titanium plates in the treatment of unilateral malar and zygomatic arch fractures Oral Surg Oral Med Oral Pathol Oral RadiolEndod 2011 112 188 194 10.1016/j.tripleo.2010.10.014 21216634

[B7] Liu XJ Gui L Mao C Peng X Yu GY Applying computer techniques in maxillofacial reconstruction using a fibula flap: a messenger and an evaluation method J CraniofacSurg 2009 20 372 377 10.1097/SCS.0b013e31819b9443 19276828

[B8] Miyamoto S Sakuraba M Nagamatsu S Hayashi R Current role of the iliac crest flap in mandibular reconstruction Microsurgery 2011 31 616 619 10.1002/micr.20929 21919049

[B9] Ghassemi A Ghassemi M Riediger D Hilgers RD Gerressen M Comparison of donor-site engraftment after harvesting vascularized and nonvascularized iliac bone grafts J Oral MaxillofacSurg 2009 67 1589 1594 10.1016/j.joms.2009.04.013 19615568

[B10] Wei R Xiang-Zhen L Bing G Da-Long S Ze-Ming T Removal of a foreign body from the skull base using a customized computer-designed guide bar J CraniomaxillofacSurg 2010 38 279 283 10.1016/j.jcms.2009.07.006 19683935

[B11] Roser SM Ramachandra S Blair H Grist W Carlson GW Christensen AM Weimer KA Steed MB The accuracy of virtual surgical planning in free fibula mandibular reconstruction: comparison of planned and final results J Oral MaxillofacSurg 2010 68 2824 2832 10.1016/j.joms.2010.06.177 20828910

[B12] Ayoub N Ghassemi A Rana M Gerressen M Riediger D Holzle F Modabber A Evaluation of computer-assisted mandibular reconstruction with vascularized iliac crest bone graft compared to conventional surgery: a randomized prospective clinical trial Trials 2014 15 114 10.1186/1745-6215-15-114 24716651 PMC3998950

[B13] Liu XZ Shu DL Ran W Guo B Liao X Digital surgical templates for managing high-energy zygomaticomaxillary complex injuries associated with orbital volume change: a quantitative assessment J Oral MaxillofacSurg 2013 71 1712 1723 10.1016/j.joms.2013.06.197 23911146

[B14] Mueller CK Zeiss F Mtsariashvili M Thorwarth M Schultze-Mosgau S Correlation between clinical findings and CT-measured displacement in patients with fractures of the zygomaticomaxillary complex J CraniomaxillofacSurg 2012 40 e93 e98 10.1016/j.jcms.2011.05.009 21733703

[B15] Oka K Murase T Moritomo H Goto A Sugamoto K Yoshikawa H Accuracy analysis of three-dimensional bone surface models of the forearm constructed from multidetector computed tomography data Int J Med Robot 2009 5 452 457 10.1002/rcs.277 19722285

[B16] Verhamme LM Meijer GJ Boumans T Schutyser F Berge SJ Maal TJ A clinically relevant validation method for implant placement after virtual planning Clin Oral Implants Res 2012 doi:10.1111/j.1600-0501.2012.02565.x 10.1111/j.1600-0501.2012.02565.x 22905668

